# Factors affecting the use of patient survey data for quality improvement in the Veterans Health Administration

**DOI:** 10.1186/1472-6963-11-334

**Published:** 2011-12-12

**Authors:** Elizabeth A Davies, Mark M Meterko, Martin P Charns, Marjorie E Nealon Seibert, Paul D Cleary

**Affiliations:** 1 Thames Cancer Registry, King's College London, 42 Weston Street, London SE1 3QD, UK; 2 Center for Organization, Leadership and Management Research, VA Boston Healthcare System, 150 South Huntington Avenue (152-M), Boston MA 02130, USA; 3 Yale School of Public Health, Yale School of Medicine, 60 College Street, PO Box 208034, New Haven CT 06520-8034, USA

## Abstract

**Background:**

Little is known about how to use patient feedback to improve experiences of health care. The Veterans Health Administration (VA) conducts regular patient surveys that have indicated improved care experiences over the past decade. The goal of this study was to assess factors that were barriers to, or promoters of, efforts to improve care experiences in VA facilities.

**Methods:**

We conducted case studies at two VA facilities, one with stable high scores on inpatient reports of emotional support between 2002 and 2006, and one with stable low scores over the same period. A semi-structured interview was used to gather information from staff who worked with patient survey data at the study facilities. Data were analyzed using a previously developed qualitative framework describing organizational, professional and data-related barriers and promoters to data use.

**Results:**

Respondents reported more promoters than barriers to using survey data, and particularly support for improvement efforts. Themes included developing patient-centered cultures, quality improvement structures such as regular data review, and training staff in patient-centered behaviors. The influence of incentives, the role of nursing leadership, and triangulating survey data with other data on patients' views also emerged as important. It was easier to collect data on current organization and practice than those in the past and this made it difficult to deduce which factors might influence differing facility performance.

**Conclusions:**

Interviews with VA staff provided promising examples of how systematic processes for using survey data can be implemented as part of wider quality improvement efforts. However, prospective studies are needed to identify the most effective strategies for using patient feedback to improve specific aspects of patient-centered care.

## Background

Despite a large literature describing and comparing patients' experiences of medical care, little is known about how such feedback can be used to improve patient-centered care [1-4]. Studies in Europe, Australia and the United States (US) have suggested that collecting and reporting patient survey data may lead to small improvements in experience across health systems [5-7] or within organizations [3,8], but others have identified a range of difficulties confronting organizations and health professionals when they try to understand and respond to survey data, implement interventions, and sustain change [2,9-11]. One US evaluation of a patient-centered care collaborative including eight health care organizations suggested that change was likely to require organizational strategies, engaged leadership, cultural change, and regular measurement and performance feedback [3]. An interview study of staff responding to data the UK National Health Service (NHS) Patient Survey Programme found these data were generally perceived positively, had potential as performance measures and to be used to develop patient-centered cultures [12]. A detailed review of four years of patient surveys in eight Danish hospitals suggested that staff viewed data positively when they perceived them as revealing problems that led to change and improvement [13]. A review by Shaller of patient-centered practices in leading US organizations identified likely change strategies as the development and training of leaders, internal rewards and incentives, training in quality improvement, and practical evidence-based tools for patient-centered care [14]. In the United Kingdom, Goodrich & Cornwell recently noted that although there are promising interventions, few have been independently evaluated [15].

Since the 1990's, the US Veterans Health Administration (VA) has made a large investment in the collection and feedback of data on clinical processes, outcomes, and patients' experiences as part of a program of transformational re-engineering and performance improvement [16]. This has resulted in improvements in immunization rates, cancer screening, and other prevention activities, as well in as the management of cardiovascular disease and diabetes [17,18]. Although few reports consider patient experience in detail, Young reported a 15% improvement between the years 1995 and 1999 [19], and the overall satisfaction of VA patients with hospital care was higher than for patients in the private sector in 2004 and 2006 [16,20]. Thus, we thought that VA staff would be a rich source of information regarding the effective use of survey data. Previous studies suggested to us that a combination of organizational focus and support and sustained motivation and understanding of data by staff was necessary to create change in survey results [2,3]. It also seemed likely that such complex change would take several change cycles over several years to achieve. Therefore, we conducted case studies of two VA facilities; one which had stable high scores and one with stable low scores. The specific goals and corresponding hypotheses for the study were:

1) To determine the promoters and barriers that health professionals and managers experienced when using patient survey results. Here we hypothesized that there would be support for survey data use within the VA, that teams would have needed to do much extra work to understand their data and "diagnose" the cause of poor patient experience results before acting; and that they would be driven to do this by long-term intrinsic motivation rather than by short-term external incentives.

2) To determine what types of supportive improvement strategies are necessary for implementing and sustaining patient-centered care. We hypothesized that these would be multi-stage and iterative, having effects over several years.

3) To assess whether improvements were related to support for developing patient-centered care, quality improvement structures, project management, and specific interventions. We hypothesized that improving and high performing facilities would demonstrate prior development of an organizational culture that promoted patient-centered care, including a coherent and data-driven overall quality improvement strategy supported by clinical leadership.

## Methods

Since 1994 the VA Office of Quality and Performance (OQP) has obtained veterans' reports about their hospital care using a standardized survey and protocol, the Survey of Healthcare Experiences of Patients (SHEP). During the period of the present study, the SHEP survey included a modified version of the Picker Institute inpatient survey tool [21], and consisted of 55 questions covering nine key aspects of the inpatient experience: access to care, courtesy of staff, education and information about the patient's illness, attention to patient preferences, coordination of care, emotional support, family involvement, physical comfort and preparation for discharge. Centralized patient records were used to select monthly stratified random samples of patients discharged from the medical, surgical and four other major services at each facility to receive a mailed survey. Following a modified Dillman technique, respondents received a pre-survey notification letter, and non-respondents had up to three follow-up contacts. Response rates were 55.5% in 2003-2004 [22]. The raw scores for each facility were posted as they arrived on VA internal websites, and reported quarterly with case-mix adjustment by OQP.

We focused on the emotional support dimension among surgical inpatients from 2002 to 2006 because the same SHEP survey was used over this period, and because facility scores on emotional support were generally lower than those observed on the other Picker dimensions, suggesting more opportunities for improvement. Previous unpublished analyses have indicated this dimension to be strongly associated with veterans' willingness to recommend facility care to others. We therefore considered that high emotional support scores were likely to be a good indicator of patient-centered practices within a facility.

Staff at OQP reviewed scores for emotional support reported by surgical patients at 106 VA surgical facilities in nine six-month periods from the end of 2002 until the end of 2006 and provided anonymous case-mix adjusted facility data to a second team member (MMM). Twenty-four facilities where less than 30 patients provided data in any six-month period were excluded. The average sample size per hospital across the nine data points for the remaining 82 facilities was 104 (range 36.5 to 198.7). MMM ranked these facilities by their baseline 2002 (Time 1) and final 2006 (Time 9) emotional support scores, and categorized them as "high," "medium" and "low" performing at both times. Cross-classifying facilities based on their Time 1 and Time 9 performance allowed facilities to be categorized further as stable, improving, or declining. He calculated Time 1 to Time 9 change scores for each facility and used these to differentiate among facilities within each category. This revealed that improving facilities were relatively rare but among those that moved from "low" to either "medium" or "high" performance between 2002 and 2006, change in average emotional support varied from 5.5 points to 18.5 points. There were few dramatic changes among those facilities in "medium" performing group. Our selection process was therefore influenced by these observed patterns in the data, and the need to select sites with definite trends in their scores over all nine periods and to identify comparison sites with stable scores. As a team we chose the two facilities that had improved the most, having moved from the "low" to the "high" performance group, and one stable low performer as a comparison site. We then identified two stable high performers and one stable medium performer as a comparison site. We reasoned that these sets of comparisons would likely provide us with useful insights on factors relevant to how facilities improved or sustained high performance by comparison to sites with consistently lower scores. This task was completed blind to the identity of the facilities so that it would not bias our categorization process.

### Recruitment of study facilities and staff within them

EAD approached the directors of the six facilities by email followed by telephone and two agreed. Four declined; one reported insufficient expertise in their facility about patient-centered care, one that staff were taking part in many other studies, one required additional local IRB approval, and one gave no reason. Both study facilities had more than 100 acute beds, provided general and specialty surgery and were affiliated with medical schools; one was urban, the other rural, and they were located in two different geographic regions of the country.

In each facility, directors were asked to suggest 10-15 staff members who may have been involved in responding to patient survey results. It was suggested that respondents might include directors or their deputies, service managers, clinicians, nurses, data managers or patient advocates. The two directors who agreed supplied contact details for 24 potential respondents, including several staff members carrying out the same role, for example, nursing staff and staff responsible for surveys about care areas other than surgery. The interviewer (EAD) selected only a few staff members with the same role and those involved in surgical surveys to approach, and contacted 16 respondents by email and telephone. Each potential respondent received an information sheet explaining the aims of the study and emphasizing that participation was voluntary. Eight agreed to an interview (5 out of 7 at Facility 1, and 3 of 9 at Facility 2). Respondents were an executive director from each facility, two patient advocates, and two customer service managers from Facility 1, and one ward nurse and one advanced nurse practitioner from Facility 2. They had worked in their facilities between one and 18 years, and in their current posts between nine months and five years. Individuals who agreed to participate were asked to give verbal consent to tape record the interview. To maintain confidentiality, their identity was not disclosed to other respondents or to their facility director. Study approval was obtained from the Institutional Review Board of the VA Boston Healthcare System and from the Health Research Ethics Committee of King's College London.

#### Interview Method

The interview schedule was adapted from a previous study of a quality improvement learning collaborative for patient-centered care in Minnesota [2,3]. The broad topics covered are shown in Table 1. In addition to asking about the current arrangements for responding to survey results and recent initiatives in patient-centered care, the interview attempted to reconstruct the sequence of events since 2002. This was done in an effort to identify factors that might distinguish high performing from medium or low performing facilities, or that characterize facilities that improve as opposed to remain stable over the longer term. Interviews followed a semi-structured technique described by Weiss [23], and lasted 35 to 70 minutes. All were conducted by telephone with respondents at work. Respondents were offered a copy of their interview transcript to correct if they wished, and a draft of the near final report was shared with them.

**Table 1:** Topic areas covered by the semi-structured interview schedule

The background to support and culture within the facility for developing patient-centered care in general and emotional support specifically
How survey data were received, disseminated, discussed, and understood within facilities

Arrangements for quality improvement project management within each facility

Usefulness of any resources or tools provided within the VA or outside

How decisions were made about which care processes should be selected for improvement

Strategies and interventions required for patient-centered care and any barriers discovered

Staff skills, training and incentives required for patient-centered care and any barriers discovered

Arrangements for evaluating the success of implementation and for sustaining any changes that occurred.

#### Data Analysis

The interviews were transcribed, and without knowing whether the facilities were improving, high or low performing hospitals, EAD read and coded them using qualitative framework analysis [24] for the themes of organizational, professional and data-related barriers and promoters to the use of patient survey data previously described [2,3]. MMM reviewed coded extracts for five interviews and discussed and agreed the previously identified themes, and some new ones revealed by the content analysis. An effort was made to integrate the accounts of different respondents within each facility to describe the process whereby change was attempted. For this EAD wrote a summary of each interview and presented a case study for each facility which was discussed and debated within the team. Examples of successful or promising change strategies e.g. plans made by leaders, accountability structures, new reporting or survey methods and staff training that were reported by two respondents are described. These examples together with the themes identified for promoters and barriers were considered as evidence to refute or confirm our hypotheses relating to goals 1 and 2 of the study. At the very end of the study after all themes had been coded and described, OQP staff revealed that neither facility had improving scores, but that one was a stable high performer and the other a stable low performer. Within the team we finally considered and compared practice within each facility to determine if the high performer could be distinguished from the low performer by organizational and practice characteristics (Table 2) and so reveal factors relevant to sustaining patient-centered care. The hypothesis of specific factors associated with improved scores in goal 3 could therefore not be directly tested.

**Table 2:** Summary of promoters and barriers to the use of survey data found in case studies of two VA facilities

	Facility 1	Facility 2
**Organizational promoters**	Nursing leaders had developed a patient-centered culture following external inspection in the middle of study period that was critical of care. This led to new quality improvement structures meeting JCAHO accreditation. In the last few years the Director has set up a Patient Satisfaction Committee and Customer Service Council to consider survey and other patient feedback to decide on improvement approaches and assess progress. Facility scores are regularly compared to others.	Clinical and nursing leaders had identified that patient-centered care needed to improve after comparing scores to other facilities at the beginning of the study period. This was in the context of developing many data-driven improvements in clinical care and raising the profile of quality improvement. Performance Improvement Facilitator reports comparative facility survey results to each management group. Clinicians and nurses present results to their teams and discuss them. Magnet Status (for nursing practice) has been obtained and reaccreditation requires review of all survey data.

**Organizational barriers**	Too few quality improvement staff to exploit the data fully. Lack of interest among some senior staff.	Large facility size and rapid turnover of patients and junior staff.

**Professional promoters**	Clinicians are beginning to ask for provider specific feedback. All facility staff have recently received interpersonal skills training. Survey results emailed to staff and presented around the facility.	Clinicians are involved in interpreting results, developing improvement plans and new surveys. Nursing leadership is focusing on bedside patient care tasks and encouraging leadership skills in nurses.

**Professional barriers**	Clinicians and other staff can be skeptical about results. Some staff do not have the skills for patient-centered care.	Nurses are less familiar with survey results. Some staff need to be reminded of patient-centered behaviors.

**Data-related promoters**	Quality improvement staff disseminate the OPQ survey data and teach other staff about it. There is an additional short local survey of all discharged patients. OPQ survey data is triangulated with qualitative feedback from all contacts with the patient advocacy service.	Administrative staff disseminate and present the OPQ survey data. Senior nursing staff teach others about them. There is an additional short local survey of all discharged patients. OPQ survey data is triangulated with feedback with "active conversations" with patients and "speak to the director slips".

**Data-related barriers**	OPQ quarterly data not seen as a large enough sample or representative enough.	

## Results

Analysis revealed promoters and barriers to using patient survey data that clustered under the topics of organizational, professional and data-related factors already described [2].

### Organizational Promoters

#### Developing a culture of patient-centeredness

Without exception respondents referred to the core VA mission of providing excellent care, based on strong values of service. For example,

*We want to do the best for the men and women who have served our country *(Advanced nurse practitioner, Facility 2)

Patient-centered care was seen as key to this:

*Whether it is a weekly message from the director, that goes out through our email, that continually stresses patient satisfaction and gratitude to staff for the care they are giving...at various meetings, presentations, committees, where again the leadership will emphasize that this is why we are here and to continue to encourage staff to give patient-centered care *(Patient advocate, Facility 1)

Executive staff spoke of their longstanding commitment to this area and of their constant work to develop the culture and reinforce relevant behaviors. For example,

*There is a lot of eye contact, a lot of saying 'hullo' to people, a lot of looking at people and offering assistance before being asked. I think that all of those things lead towards the patients feeling very connected to the hospital *(Director, Facility 1)

#### Developing quality improvement structure and skills

Respondents described structures for routinely reviewing survey feedback. At Facility 1 a monthly Patient Satisfaction Committee reviewed these data and other feedback. Progress and problems were reported to a quarterly Customer Service Council where executive leadership would request investigations and suggestions for solutions. A customer service manager disseminated results to the service chiefs for medicine and surgery. In Facility 2, a performance improvement coordinator reported survey results to the management group of each clinical unit. Supervising physicians and nursing managers explained the results directly to other frontline staff and encouraged their involvement in finding solutions.

#### Persistence of quality improvement staff

Respondents were aware that VA clinical care had improved and that excellence was sought in all areas.

*I do know that the VA, and our VISN (Veterans Integrated Service Network; the VA regional structure), and our facility are very interested in meeting, not only meeting but exceeding the patient's expectation... it's just a way of doing business*. (Customer service manager, Facility 1)

Respondents in Facility 2 described examples of quality improvement projects in preventing falls, hospital infection, post operative complications, and adverse drug events, and how lessons from these projects were applied to improving patient experience. Senior staff in both facilities described the importance of avoiding complacency. For example,

*Continuously taking in information and looking ahead to where you are going for focus groups and getting feedback and being prepared for the future, while you are taking care of your present customers. So I would say moving towards more of a learning culture*. (Customer service manager 2, Facility 1)

#### External comparison, accountability, and audit

Feedback sources external to the facility were a new theme described as important. The comparative survey data provided by OQP meant that directors were aware of the areas their facilities "fell down in," and efforts to improve emotional support in particular were cited as resulting from this attention. Another mechanism driving improvement efforts was a VA inspection that followed a complaint related to issues at Facility 1 that had been reflected in survey feedback, but not acted upon. As a result, the facility strengthened its focus on patient-centered care, and senior nurses set up a quality improvement structure linked to a national accreditation process.

#### Learning from other facilities and organizations

Respondents in Facility 1 also mentioned learning about good practice from other VA facilities and non-VA organizations. These activities depended on individuals making contacts, but the point was made that top-down approaches were not enough:

*While a national organization can set a vision, it needs to really reinforce that the improvements, and the celebration of those improvements, need to be at the local level and that incentive for that improvement and for testing out those improvements need to be there*. (Director, Facility 1)

For example, Facility 2 had achieved Magnet Status, [25] and the revalidation process every four years provided an impetus to review all patient survey data and related nursing practice; patient-centered care became a matter of local pride and was seen as providing a competitive edge.

### Organizational Barriers

Organizational barriers mentioned included increased patient volume, large facility size, and rapid turnover of junior medical staff, lack of interest among some other senior executive staff, and too few quality improvement staff to exploit the data fully. Interviewees reported that all of these factors made it difficult to find the time to thoughtfully review patient satisfaction feedback and engage medical staff in improvement efforts.

### Professional Promoters

#### Clinical leadership

Due to reported lack of time and greater interest in evidence-based care, physicians were described as "partners" or "participants" rather than leaders in improving patient-centered care. Physicians were involved in interpreting results, developing improvement plans and designing questions for shorter surveys. At Facility 1 the director had made sure that physicians had comparative data on their practice, and gave each chief of service responsibility for understanding data and making changes.

#### Selecting and training staff in people skills

It was recognized that staff required particular skills to provide patient-centered care. Facility 1 trained every staff member from executive to frontline staff to improve their interpersonal skills in taking responsibility for responding to patients' needs. For example clerical and grounds staff were trained to bring patients' confusion or distress to the attention of clinical staff.

#### Structured feedback of results to staff

In addition to presenting survey results in meetings with frontline staff, each facility distributed them by email. Facility 2 publicized the findings in posters around the site, and included photographs of staff with patients.

#### Nursing leadership

A new theme emerging in both facilities was that the nursing executive played a major role in implementing patient-centered initiatives. Nurses described this as listening carefully to veterans, understanding their diverse and often complex physical and emotional needs, making sure things "got taken care of," and being willing to change their behavior. Facility 2 had made significant efforts to move nursing care away from charting tasks and "back to the bedside." Nurse supervisors led frontline staff by example, closely checking that patients were comfortable, felt supported and that their symptoms were controlled. At the same time, junior nurses were encouraged to develop leadership skills by becoming involved in committees for the governance and quality improvement of care within their units, and presenting improvement results at conferences.

### Professional Barriers

#### Clinical skepticism

Several non-physician respondents thought that physicians were more skeptical of survey data than other staff, but in general skepticism was viewed as a personality rather than a generic professional issue. The challenge of teaching frontline staff to understand survey data as relevant to their practice was acknowledged.

#### Defensiveness and resistance to change

Respondents reported familiarity with the problem of some employees being unwilling to change.

*Well there are the words: "I have never had to do it before, why should I have to do it now? I have been here 35 years. I am not going to do it. I'll retire first". That is the government way - you can't make me do it*. (Patient advocate, Facility 1)

#### Lack of staff selection, training or support

The fact that not all staff had the skills necessary to improve patient-centered care was also acknowledged.

*The challenge is that a lot of people don't have awareness - maybe a realistic awareness of how they impact on others. They might say "Oh, you know, this is common sense" and although it may be common sense, it might not be common practice" *(Customer service manager 2, Facility 1)

### Data-Related Promoters

#### Expertise with data

Both facilities employed administrative or quality improvement staff to disseminate and explain the data. Each also reviewed the survey questions to understand what might contribute to performance. At Facility 2 the nursing executive described teaching nurses about the questions patients were asked so they knew how their care would be evaluated.

*I say to them you don't want to get a poor report card just because you forgot to get something for the patient*. (Nursing executive, Facility 2)

#### Timely feedback of data

Both facilities supplemented the results received centrally with local data collected at more frequent intervals, including a shorter questionnaire given to all patients discharged from the facility.

#### Triangulation of different data sources

A further new theme emerging from these interviews was the practice of staff collecting additional data to further understand patients' perspectives. These included "active conversations" by staff with patients in their daily practice, "speak to the director" feedback slips, and a daily "how did we do today" questionnaire. Facility 1 had recently invested in recording details of issues patients raised in person with patient advocates. A Patient Advocate Tracking System was used to code problems using the SHEP survey categories, send departments a request that they be solved, track problems over time and report the top five issues to the Patient Satisfaction Committee and Executive Team each month.

### Data-Related Barriers

#### Lack of timely feedback

Some staff at Facility 1 did not think that the official, case-mix adjusted quarterly survey reports were timely enough to stimulate change and this led to the emphasis on collecting additional data.

#### Lack of specificity and discrimination

A related issue was that a sample of 30 patients was not seen as providing sufficiently specific data to act on.

*You need a larger sample to break down the resistance: It's not me; it's not my patient *(Director, Facility 1)

#### Uncertainty about effective interventions

Although respondents thought that a wide range of activities were necessary to improve patient experience, none were entirely sure which specific interventions would improve survey scores. Results were reported as "improved" and problems reported as being detected using new data collection systems. However, no respondent described a specific intervention having been shown to improve scores on emotional support. Identifying effective interventions and sharing these within the VA was generally expressed as a need to be met.

### Deducing Facility Performance Status From Interview Data

After all interpretation of the interviews and observations were completed, we compared the promoters in each facility and the timing of major initiatives during the study period (Table 2). We first estimated which facility we thought was the stable high performer and which was the stable low performer on emotional support. We judged that Facility 2, with its longer standing quality improvement system, engagement of clinical and nursing leaders and consistent ownership of results and generation of potential solutions was likely to be the stable high performing facility. However, this turned out not to be the case; Facility 1 was the stable, high performer.

## Discussion

This study used interviews with staff at two VA facilities to assess organizational, professional and data-related promoters and barriers to the use of patient survey data in quality improvement. It also attempted to use these interview data to develop case studies of practice over time and to deduce facility performance status on emotional support. In relation to the first study goal of determining barriers and promoters we found evidence at both facilities for our first hypothesis of support within the VA for survey data use. Respondents described many ways in which the use of survey data was promoted, and fewer examples of barriers than described previously in organizations with less experience of using this data [2,3]. A new organizational theme emerging was the impetus to change provided by comparative results for VA facilities and by directors' accountability for patient-centered care. Although physicians were described as supportive of improvement efforts, we found less evidence of leadership by them. Instead, nursing leadership for quality improvement and patient-centered care emerged as a key new theme to promoting change. Our second hypothesis that teams would need to do additional work to understand their data and "diagnose" the causes of poor patient experiences was supported. VA staff described efforts to raise the profile of the survey data among staff, to increase their familiarity with and understanding of these data, and to collect additional quantitative and qualitative data and triangulate this new information with the SHEP data. Although staff appeared highly motivated and to be actively involved in developing patient-centered cultures thereby supporting our third hypothesis, it was not possible using this study design to identify or isolate the effects of any multi-stage and iterative improvement strategies over several years. Respondents were enthusiastic about trying out new interventions, but could provide few examples of documented links between particular changes in care and shifts in survey scores for emotional support. In respect of the second goal of the study we found some evidence of the types of support likely to be necessary for implementing and sustaining patient-centered care including the importance of leaders being motivated to develop an organizational culture that gave this priority, and of leadership linked to a coherent overall quality improvement strategy, but we could not directly test our fourth and fifth hypotheses on these topics. We made intensive efforts to understand the specific kinds of policies and activities that facilitate and/or inhibit patient-centered care, but the data did not allow us to achieve this third goal of the study. Although we identified many factors that we thought were likely to be related to how well the facility provided such care, in the final analysis we made the wrong judgment about these differences in terms of distinguishing whether their performance was high or low. With the benefit of hindsight and un-blinded knowledge of actual performance, clues about lower emotional support in the second facility can be seen in interviews describing continued attempts to improve this element of patient experience. On reflection, there were insufficient data from individuals with experience spanning the full study period; information from more such individuals could have made the decision more accurate.

### Limitations

Only two of the six facilities approached agreed to participate and only half of the potential respondents approached agreed to be interviewed. Thus, the findings may not be representative of practices at other facilities and did not allow us to compare a range of large and small hospitals and explore why the latter tend to have better patient experience scores. The study facilities may also be those where patient-centered care was salient to the director and the staff who agreed to interview may have wanted to report their success and interest. Although negative factors and experiences were described, it is also possible that respondents felt uncomfortable describing barriers and less positive accounts to a non-US interviewer. However, data obtained from different respondents at each facility did appear consistent, although directors' accounts of their motivation and strategy for beginning patient-centered initiatives could not be cross-checked.

### Comparison with other findings

There are few published evaluations of methods of using patient experience data to improve patient-centered care with which to compare our findings [4,15]. However, two studies have recently reported on the use of data from large survey programs. Reeves and Seccombe [12] interviewed patient survey leads from each of 24 English hospitals about the NHS national patient survey programme. So far there have been few improvements in patient experience except in areas of government initiatives, but these authors found broadly similar results in that the data were being used to plan improvements and were generally perceived positively among NHS staff, particularly as surveys were repeated more often and provided clear comparative information and qualitative comments from patients. Other promoters of data use similar to those found here included the development of a patient-centered organizational culture, and the incentive provided by including patient survey data in performance measures. Similar barriers included data not being considered specific enough for particular units or departments, and lack of resources and expertise to use the data fully as evinced in our study by descriptions of, respectively, local attempts to collect additional local data on all discharged patients and some comments about too few quality improvement staff. Riiskaer and colleagues [13] reported a study of eight Danish hospitals where four consecutive patient surveys had been performed over seven years. Although these authors were careful not to attribute improved patient experience over the study period to the surveys, they found that improvement had occurred particularly in wards that initially had poor scores. Managers tended to be more positive about survey data than clinicians, and those in wards where patient experience had improved were more positive about the data than those whose results stayed the same [13]. As was true in the present study, Riiskaer and colleagues found general acceptance of surveys as a tool for improvement, clinicians' need for specific diagnostic information at unit level, and the value that staff found in reading accompanying qualitative comments. However, our study is the first to focus on a large organization which has established a systematic process over many years for feeding back quarterly comparative survey data to staff across many hospitals. New promoters for using survey data included a range of external incentives provided within the VA by these data and the important of senior nursing leadership in making practice changes. The VA facilities we studied also provided some promising examples of strategy, programs and interventions used to develop patient-centered policies. Although interviewees identified fewer barriers to using data, they did identify the need to change staff behaviors as a result of it. Finally our study uncovered some unexpected methodological problems associated with studying hospitals with differing performance that future studies of the specific processes and interventions needed in this field may now avoid.

### Implications for future research and practice

We used a unique longitudinal database of patient experience data to identify VA facilities with objective and sustained evidence of high performance or improvement for more detailed study. This research strategy might be employed using this VA database or others from large health care systems such as the NHS. However, experience from this study suggests the need for a range of fieldwork methods in future studies. In retrospect, site visits by a team of researchers, face to face interviews, and the ability to follow up other respondents and to source facility documents and policies might have led to a more detailed understanding of the history of change processes at any facility. Nonetheless, this telephone study provided promising examples of how systematic processes for using patient experience survey data can be implemented as part of a wider quality improvement structure. Prospective studies might also examine a number of facilities using patient experience data to measure improvements in specific aspects of patient-centered care as a result of defined interventions. We suggest that higher profile multi-site studies of the implementation of initiatives offering training and support [3] might also encourage directors to agree to their facilities being included. Studies to date have not reported whether patients were directly involved in the quality improvement process [26] and this strategy should be evaluated. Using qualitative data in combination with survey data would also seem an appealing strategy to test in delivering sustained change in patient experience.

## Conclusions

Interviews with VA staff provided promising examples of how systematic processes for using survey data can be implemented as part of wider quality improvement efforts. Prospective studies are needed to identify the most effective strategies for using patient feedback to improve specific aspects of patient-centered care in the VA and more widely.

## Competing interests

The authors declare that they have no competing interests.

## Authors' contributions

EAD conceived and designed the study, helped analyze the survey data, collected and interpreted the interview data, and wrote the paper. MMM helped design the study, analyzed the survey data, helped interpret the interview data and write the paper. MPC and PDC helped to design the study, select the study facilities, interpret the interview data and revise the paper. MENS helped to design the study, interpret the interview data and revise the paper. All authors read and approved the final manuscript.

## Pre-publication history

The pre-publication history for this paper can be accessed here:

http://www.biomedcentral.com/1472-6963/11/334/prepub

## References

[B1] Cleary PD Edgman-Levitan S Walker JD Gerteis M Delbanco TL Using patient reports to improve medical care: a preliminary report from ten hospitals Qual Manage Health Care 1993 2 1 31 38 10.1136/qshc.2.1.31 10131018

[B2] Davies E Cleary PD Hearing the patient's voice? Factors affecting the use of patient survey data in quality improvement Qual Saf Health Care 2005 14 6 428 432 10.1136/qshc.2004.012955 16326789 PMC1744097

[B3] Davies E Shaller D Edgman-Levitan S Safran DG Oftedahl G Sakowski J Cleary PD Evaluating the use of a modified CAHPS survey to support improvements in patient-centred care: lessons from a quality improvement collaborative Health Expect 2008 11 2 160 176 10.1111/j.1369-7625.2007.00483.x 18494960 PMC5060434

[B4] Coulter A Fitzpatrick R Cornwell J Measures of patients' experience in hospital: purpose, methods and uses 2009 London: The King's Fund http://www.kingsfund.org.uk/publications/measures.html

[B5] Draper M Cohen P Buchan H Seeking consumer views: what use are results of hospital patient satisfaction surveys? Int J Qual Health Care 2001 13 6 463 8 10.1093/intqhc/13.6.463 11769748

[B6] Gilles RR Shortell SM Casalino L Robinson JC Rundall TG How different is California? A comparison of US physician organizations Health Affairs 2003 Millwood http://content.healthaffairs.org/cgi/reprint/hlthaff.w3.492v1.pdf 10.1377/hlthaff.w3.492 15506154

[B7] Richards N Coulter A Is the NHS becoming more patient-centered? Trends from the national surveys of NHS patients in England 2002-07 2007 Oxford: Picker Institute http://www.pickereurope.org/Filestore/PIE_reports/project_reports/Trends_2007_final.pdf

[B8] Hildenhovi H Nojonen K Laippala P Measurement of outpatients' views of service quality in a Finnish university hospital J Adv Nurs 2002 38 1 59 67 10.1046/j.1365-2648.2002.02146.x 11895531

[B9] Rogut L Hudson A Meeting patients' needs: quality care in a changing environment 1995 New York: United Hospital Fund of New York, Paper Series 10164376

[B10] Tasa K Baker R Murray M Using patient feedback for quality improvement Qual Manage Health Care 1996 4 55 67 10.1097/00019514-199600420-00008 10154537

[B11] Wensing M Vingerhoets E Grol R Feedback based on patient evaluations: a tool for quality improvement? Patient Educ Couns 2003 51 149 53 10.1016/S0738-3991(02)00199-4 14572944

[B12] Reeves R Seccombe I Do patient surveys work? The influence of a national survey programme on local quality-improvement initiatives Qual Saf Health Care 17 437 441 10.1136/qshc.2007.022749 PMC2602592 19064659

[B13] Riiskjaer E Ammentorp J Nielsen JF Kofoed P-E Patient surveys - a key to organizational change? Patient Educ Couns 2010 78 3 394 401 Epub Oct 2 10.1016/j.pec.2009.08.017 19800757

[B14] Shaller D Patient-Centered Care: What does it take? 2007 New York: Commonwealth Fund http://www.commonwealthfund.org/Content/Publications/Fund-Reports/2007/Oct/Patient-Centered-Care--What-Does-It-Take.aspx

[B15] Goodrich J Cornwall J Seeing the person in the patient The Point of Care review paper 2008 London: The King's Fund http://www.healthissuescentre.org.au/documents/items/2010/05/319001-upload-00001.pdf

[B16] Perlin JB Kolodner RM Roswell RH The Veterans Health Administration: quality, value, accountability and information as transforming strategies for patient-centered care Am J Managed Care 2004 10 part 2 828 836 15609736

[B17] Jha AK Perlin JB Kizer KW Dudley RA Effect of the transformation of the Veterans affairs health care system on the quality of care NEJM 2003 348 2218 2227 10.1056/NEJMsa021899 12773650

[B18] Kerr EA Fleming B Making performance indicators work: experience of US Veterans Health Administration Br Med J 2007 335 971 973 10.1136/bmj.39358.498889.94 PMC2072029 17991979

[B19] Young GJ Managing organizational transformations: Lessons from the Veterans Health Administration Calif Manage Rev 2000 10 224 8

[B20] US Department of Veterans Affairs Veterans' health care outscores private sector- again 2006 http://www1.va.gov/opa/pressrel/pressrelease.cfm?id=1069 Press release

[B21] Cleary PD Edgman-Levitan S Roberts M Moloney TW McMullen W Walker JD Delbanco TL Patients evaluate their hospital care: a national survey Health Aff 1991 10 4 254 267 10.1377/hlthaff.10.4.254 1778560

[B22] Wright S Craig T Campbell Schaefer J Humble C Patient satisfaction of female and male users of Veterans health administration services J Gen Internal Med 2006 21 1525 1547 10.1111/j.1525-1497.2006.00371.x PMC1513173 16637941

[B23] Weiss R Learning from strangers: the art and method of qualitative interview studies 1994 New York: Free Press, Maxwell Macmillan International

[B24] Ritchie J Spencer L O'Connor W Ritchie J, Lewis J Carrying out qualitative analysis Qualitative Research Practice: A Guide for Social Science Students and Researchers pp 219-262 2003 London: Sage Publications

[B25] American Nurses Credentialing Centre http://www.nursecredentialing.org/Magnet.aspx

[B26] Sweeney G O'Hagan B Squire S Powell C The patients accelerating change project: does it make a difference? Clinical Governance: An International Journal 2005 10 1 72 83 10.1108/14777270510579323

